# Cell type-specific genotoxicity in estrogen-exposed ovarian and fallopian epithelium

**DOI:** 10.1186/s12885-020-07524-7

**Published:** 2020-10-21

**Authors:** Liang Song, Zizhi Tang, Changsheng Peng, Yueming Yang, Chang Guo, Danqing Wang, Liandi Guo, Jie Chen, Cong Liu

**Affiliations:** 1 Key Laboratory of Birth Defects and Related Diseases of Women and Children (Ministry of Education), Department of Gynecology, Meishan Women and Children’s Hospital, West China Second University Hospital, Sichuan University, Chengdu, 610041 People’s Republic of China; 2 College of Pharmacy, Southwest Minzu University, Chengdu, 610041 People’s Republic of China

**Keywords:** Ovarian surface epithelium, Steroid hormone, DNA damage, Homologous recombination

## Abstract

**Background:**

Loss of the genomic stability jeopardize genome stability and promote malignancies. A fraction of ovarian cancer (OvCa) arises from pathological mutations of DNA repair genes that result in highly mutagenic genomes. However, it remains elusive why the ovarian epithelial cells are particularly susceptible to the malfunction of genome surveillance system.

**Methods:**

To explore the genotoxic responses in the unique context of microenvironment for ovarian epithelium that is periodically exposed to high-level steroid hormones, we examined estrogen-induced DNA damage by immunofluorescence in OvCa cell lines, animal and human samples.

**Results:**

We found that OvCa cells are burdened with high levels of endogenous DNA damage that is not correlated with genomic replication. The elevation of damage burden is attributable to the excessive concentration of bioactive estrogen instead of its chemomimetic derivative (tamoxifen). Induction of DNA lesions by estrogen is dependent on the expression of hormone receptors, and occurs in G1 and non-G1 phases of cell cycle. Moreover, depletion of homologous recombination (HR) genes (*BRCA1* and *BRCA2*) exacerbated the genotoxicity of estrogen, highlighting the role of HR to counteract hormone-induced genome instability. Finally, the estrogen-induced DNA damage was reproduced in the epithelial compartments of both ovarian and fallopian tubes.

**Conclusions:**

Taken together, our study disclose that estrogen-induced genotoxicity and HR deficiency perturb the genome stability of ovarian and fallopian epithelial cells, representing microenvironmental and genetic risk factors, respectively.

**Supplementary information:**

**Supplementary information** accompanies this paper at 10.1186/s12885-020-07524-7.

## Background

Cancer genomes accumulate mutations upon loss of genome stability. During carcinogenesis and progression of cancer, cells are challenged by various genotoxic insults, which pose threats to genomic stability [1, 2]. Encounter of DNA lesions requires efficient DNA damage responses (DDR) to prevents loss and gain of genetic information. DNA lesions can be quickly monitored by ATM (Ataxia-Telangiectasia mutated) and ATR (A-T-related)-mediated cell cycle checkpoint [3], followed by actions of repair factors such as RAD51, BRCA1 and 53BP1 to eliminate DNA breaks. For DNA double strand breaks (DSBs) that generated frequently during genomic replication, appropriate repair pathways including non-homologous end joining (NHEJ) and homologous recombination (HR) are activated in G1 and S/G2 phases, respectively [4–6]. For HR, RAD51 is the essential recombinase, whose recruitment to DNA breaks is dependent on RPA-coated ssDNA (single-strand DNA) filaments and HR mediator factors (ie. BRCA2) [7, 8].

Loss of DDR function via deleterious mutation impair genomic stability, and consequently lead to high-frequent point mutations and structural variation at chromosomal level, which eventually promote carcinogenesis [9, 10]. For example, pathogenic mutations (ie. *BRCA1, BRCA2*) that debilitates HR are frequently associated with increase of cancer predisposition, manifested in 17.1% of the familial and sporadic breast cancer patients [11–13]. Similarly, ~ 50% cases of high-grade ovarian cancers carry *BRCA1* and *BRCA2* mutations, causing high-incidence of homologous recombination deficiency (HRD) [14–16]. Although HR mutations, including those arisen from familiar and hereditary sources, affect all types of proliferating cells, risk of malignancies are mainly limited in tissues like breast, ovary and prostate [17, 18]. Up to date, it remains mysterious why the epithelial compartments in these tissues are uniquely susceptible to carcinogenesis upon HR ablation.

Steroid hormones including estrogen, progestogen, androgen, and possibly their derivatives, are genotoxic to ovarian epithelial cells that expressing hormone receptors [19]. Activation of hormone receptors (ie. ER) can induce DSBs and require Topoisomerase II (TOP2) to resolve decatenated DNA. It was reported that endogenous DNA lesion is transiently elevated in G1 phase of breast cancer cells upon increased concentration of estrogen [20]. BRCA1 and endonuclease activity of MRE11 is obligated to remove TOP2 adducts-associated DSBs. Although this model points to the resolution of estrogen-induced TOP2 adducts, and can be expanded to ovarian epithelium whose microenvironment is periodically flushed with even higher concentration of estrogen [21], it does not thoroughly explain the susceptibility of breast/ovarian epithelium to other HR mutations (ie. *BRCA2*), which functions in S/G2 phase instead of G1. Moreover, the genomic footprints in *BRCA1*-mutated breast cancer are distinct from those bearing *BRCA2* mutations, implying the involvement of different mutational processes/mechanisms [22].

Here, we hypothesized that estrogen-induced genotoxicity contributes to the tissue susceptibility of ovarian epithelium to HRD. We characterized the DDR patterns using cell lines, clinically derived ovarian cancer tissues as well as mouse models. We show that arising of DNA breaks are independent of proliferation of OvCa cells, and thus irrelevant to erroneous genomic DNA replication. Instead, high-level estrogen contributes to the accumulation of DNA lesions in ER-positive OvCa, which act in both G1 and non-G1 phases of cell cycle. ER-induced genotoxicity requires functional HR. At last, estrogen-induced genotoxicity was rigorously reproduced in non-cancerous epithelial compartment of ovary and fallopian tubes. Altogether, we conclude that estrogen specifically challenge the genomic integrity in ovarian epithelium. This demands the functional HR to curb the carcinogenesis of ovarian cancer.

## Methods

### Cell culture

Cancer cell lines were grown in Dulbecco’s DMEM supplemented with 10% calf serum plus 100 U/ml Penicillin and 100 mg/ml streptomycin. SKOV-3 (HTB-77) and A549 (CCL-185™) were purchased from ATCC. HO8910 (3111C0001CCC000280) and HO8910-PM (3111C0001CCC000281) were purchased from National Infrastructural of Cell Line Resources, Beijing. OVCAR-8TR and L02 lines were gifted by collaborating laboratory. Cell lines were authenticated upon purchase and regularly tested for mycoplasma contamination.

### Biopsies, cryosections and ex vivo culture of ovarian tissue

Ovarian tissues diagnosed with OvCa were used in this study along with one ‘healthy’” ovary without detectable malignancy. Freshly excised tissue blocks were embedded in OCT, followed by snap frozen in liquid nitrogen and stored in -80 °C. Deep frozen tissue blocks were sectioned at 8 μm using microtome (LEICA, CM3050 S). For ex vivo culture of OvCa biopsies, freshly excised tissues were subjected to mincing and enzymatic digestion (Collagenase A, Roche, 10,103,578,001; Trypsin, GIBCO, 25200–056) for 2 h with occasional vortex. Isolated single cells were dispersed in RPMI-1640/10% FBS and grown in media supplemented with 10 mg/ml Matrigel (BD, 356234). After 48 h, experiments were performed when 70% of cells were attached.

### In vivo experiments

Eight reproductively mature female mice (C57/B6, 6 weeks old) were divided into two groups: group A, no estrogen treatment; group B, intraperitoneal injection with estrogen (Selleck, S1709, 1 mg/kg) for 6 h. Dissected liver, ovary and fallopian tube were instantly frozen in liquid nitrogen and cryosectioned for indirect immunofluorescent staining. All animals were housed in standard SPF condition throughout the experiments. Animals were sacrificed with minimal pain by neck broken protocol following approved CO2 euthanasia procedure.

### Immunostaining and fluorescence microscopy

Cryopreserved sections or cells grown on coverslip were fix with 4% paraformaldehyde (PFA) and permeabilize with 0.3%TritonX-100, followed by blocking in PBS with 3%BSA, 3% donkey serum and 0.2% Triton X-100. Primary antibodies were diluted with antibody buffer (3% Triton/10% BSA in PBS) and incubated overnight: phoshpo-BRCA1 (Bethyl, A300-001A, 1:1000), phosphor-RPA32 (NOVUS, NB100–544,1:3000), RAD51 (Proteintech, 14,961–1-AP, 1:500), Cyclin A2 (Huabio, ET-1612-26,1:500), anti-53BP1 (Bethyl, A300-272A,1:1000), anti-γH2AX (Millipore, 05–636,1:500), anti-BRCA2 (Invitrogen, MA5–32986, 1:500). Fluorescent images were acquired using OLYMPAS (BX51) and images were processed analysed using Image-Pro Plus software.

### Chemical treatments

Cells were treated with hormones at the following concentrations: tamoxifen (Sigma, T5648), 50 nM; estrogen (17β-estradiol, Wako), 50 nM. Intraperitoneal injection of estradiol (Selleck, S1709, 1 mg/Kg) was applied to animals. Time of treatment are described for each experiment.

### RNA interference and RT-PCR

Lipofectamine 3000 transfection kit (Invitrogen, 3,000,015) was used for siRNA transfection for cells grown to 70% confluency. Cells were collected 48 h after transfection for indicated experiments. Individual siRNA duplexes used were: BRCA1 (target sequence: UCUGCUGUAUUGGAACAAAUU); BRCA2 (target sequence: AAC AACAAUUACGAACCAAACUU). Knockdown efficiencies of these genes are shown in Supplementary Figure 2. To quantify gene expression levels, total cellular RNA was extracted by using TRIzol® Reagent (Invitrogen) and cDNA was synthesized using Eastep RT Master Mix Kit (Promega). RT-PCR products were visualized by agarose gel electrophoresis. mRNA levels were normalized using GAPDH or actin RNA as internal control.

### Statistical analysis

The Student’s *t*-test was performed on all data analysis. Each experiment had at least three independent biological replicates. Unless otherwise specified, data are showed as mean ± s.e.m. *p* < 0.05 was considered to be statistically significant. Excel and GraphPad Prism was used to create the graphs and calculate the *p* value.

## Results

### Endogenous DNA damage in OvCa cells is not correlated with genomic replication

**Fig. 1:**
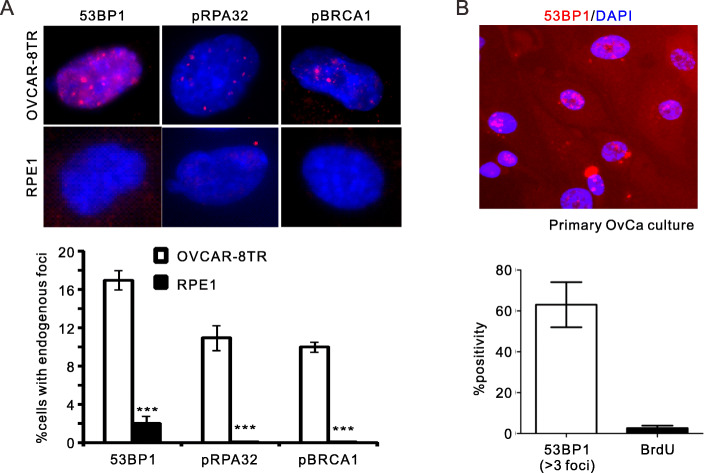
Uncoupled elevation of DNA damage and genomic replication in ovarian cancer cells. **a**, Indirect immunofluorescent (IF) staining of endogenous foci formation of indicated repair factors in OVCAR-8TR and RPE1 cells (upper panel) and quantifications for positive cells (lower panel). 53BP1, RPA32-pSerine 33 and BRCA1-pSerine1524 were monitored by specific antibodies. ***: *p* < 0.001, two-tailed unpaired *t*-test. Three biological replicates were analyzed. **b**, Representative images (upper) and quantification (lower) for 53BP1-positive cells (> 3 per cell) in primary cells isolated from three independent OvCa biopsies after culturing in RPMI1640/Matrigel for 48 h. BrdU-positive cells were quantified in parallel. Data showed as means ± SEM of 3 independent assays

In advanced stages of cancer, uncontrolled DNA replication in fast-proliferating cancer cells can increase the chances of DNA damage. To evaluate the level of endogenous DNA damage in OvCa cells, we examined the activation of DSB repair factors in OVCAR-8TR cells. In comparison with hTERT-transformed human primary cells (RPE1, non-cancerous) without obvious DNA breaks, ~ 11–17% percentage of OVCAR-8TR cells were marked with foci of 53BP1, phosphorylated RPA32 and BRCA1 (Fig. 1a), indicating significant levels of endogenous DNA damage in OvCa cells.

Endogenous DNA breaks in OvCa cells were further solidified by assays in ex vivo culture from fresh surgical biopsies. We found 63% primary OvCa cells displayed endogenous 53BP1 foci (> 3 per cell) (Fig. 1b). However, it seems that the basal level of 53BP1 was not correlated with the genomic replication in ex vivo culture, as manifested by the low rate of BrdU-positive OvCa cells (2%). Thus, we conclude that OvCa cells suffer from strong genotoxic burden that is not accompanied by genomic replication.

### Estrogen triggers DNA breaks in OvCa cells

Ovarian surface epithelium is periodically exposed to high level of steroid hormones (estrogen and progesterone), triggering cycles of proliferation, damage and repair known as ovulation trauma [23]. Besides potent proliferation stimulation, estrogen also has a direct genotoxic impact on estrogen-positive cells (ie. breast), albeit the precise mechanism remains elusive [24–26]. We hypothesized that the high level of endogenous DNA lesions observed in above OvCa cells, including a significant fraction of from replication-independent damage, may largely arise from exposure to estrogen containing in culturing media.

**Fig. 2:**
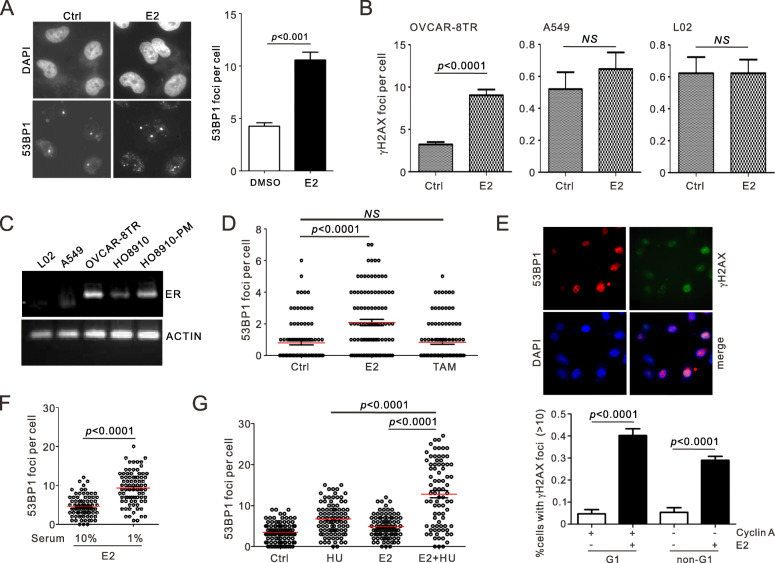
Estrogen induces DNA damage in both G1 and S phases of OvCa cells. **a-b**, Indicated cells were pre-treated with estrogen (E2, 50 nM) for 12 h. DSBs were detected by IF staining of 53BP1 (**a**) and γH2AX (**b**). Estrogen triggers elevation of endogenous DNA damage in OVCAR-8TR instead of A549 and L02 cells. **c**, Comparison of *ER* gene expression by semi-quantitative PCR in indicated cell lines. The images were cropped and full-length gels are presented in Supplementary Figure 1. **d**, Parallel quantification of 53BP1 foci in OVCAR-8TR cells pre-treated with estrogen (E2) or tamoxifen (TAM, 50 nM) for 3 h. There was no DSB induction by TAM. **e**, Quantification of 53BP1 foci in cyclin A-positive (G1) and negative (non-G1) cells, respectively. **f-g**, Enumeration of E2-induced 53BP1 foci in OVCAR-8TR cells pre-treated with serum starvation for 24 h (**f**) or hydroxyurea (**g**) for 2 h (**g**). Statistic analysis in this Figure: *p* values were calculated by unpaired *t*-test between two groups in 3 biological repeats; *NS*: non-significant

To determine the impact of steroid hormones on the origin of DNA lesions in ovarian cells, estrogen was added to proliferating OVCAR-8TR cells, followed by the monitoring of 53BP1 and γH2AX-represented DSBs. Clearly, addition of 50 nM estrogen for 12 h elevated the endogenous level of 53BP1 and γH2AX foci, indicating a robust generation of DNA breaks upon steroid stimulation (Fig. 2a-b). These hormone-induced DNA lesions were correlated to the expression levels of estrogen nuclear receptor (ER), as cells originated from non-female reproductive tissues including L02 (liver) and A549 (lung cancer) displayed minimal basal level of γH2AX (< 1 per cell), and did not respond to estrogen treatment (Fig. 2b). Coherently, expression levels of ER in OvCa cells (OVCAR-8TR, HO-8910 and HO-8910 PM) as measured by RT-PCR were appreciably higher than L02 and A549 cells (Fig. 2c). Furthermore, addition the same concentration of tamoxifen, which is the chemical mimics of estrogen but biologically inactive, did not induce equivalent number of 53BP1 foci in OVCAR-8TR cells (Fig. 2d). Thus, we conclude that exposure of ER-positive OvCa cells to estrogen triggers genotoxicity.

### Estrogen-induced genotoxicity in different cell cycle phases

Sasanmura et al. detected DSBs in G1 phase upon estrogen induction20, in which stage HR is not active due to the absence of homologous sister chromatids. We investigated if the estrogen-dependent elevation of 53BP1 occurs in cell cycle phases beyond G1. Although previous work described foci formation after treatment with 10 nM estrogen for 2 h in MCF-7 cells, DNA lesions were not detectable shorter than 6 h in OVCAR-8TR cells. In asynchronized OVCAR-8TR culture, γH2AX increase (> 10 foci per cell) was observed in 40% of cyclin A-positive cells, which represents G1 phase cells (Fig. 2e). This is consistent to the estrogen-induced accumulation of 53BP1 foci in serum starved OVCAR-8TR cells (Fig. 2f), confirming the impact of estrogen on G1 cells. In parallel, we also detected comparable elevation of 53BP1 in a subpopulation of cyclin A-negative cells (29%), indicating that non-G1 phase cells (S/G2 and mitosis) are also liable to the genotoxicity of estrogen (Fig. 2e). Consistently, arresting cell cycle in S phase by pre-treating OVCAR-8TR with hydroxyurea produced a remarkable additive elevation of 53BP1 foci on top of estrogen exposure, indicating that S-phase cells are sensitive to estrogen (Fig. 2g). Thus, estrogen-induced genotoxicity can occur in both G1 and S phases of OvCa cells, implying the involvement of HR-dependent and independent mechanisms to eliminate these damages.

### Activation of HR upon estrogen exposure

**Fig. 3:**
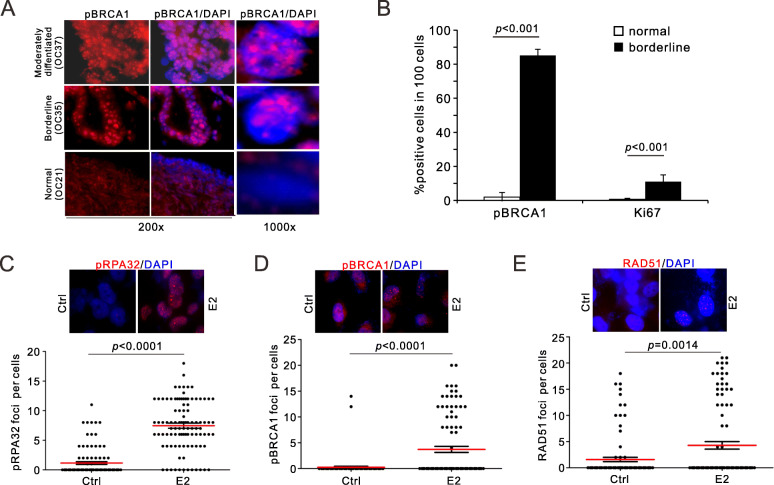
DSB recruitment of HR proteins upon estrogen induction. **a-b**, IF staining (**a**) and quantification (**b**) for phosphor-BRCA1 in cryosectioned ovarian cancer at indicated stages. *p* values were calculated between two indicated groups by unpaired t-test. *p* values: unpaired *t*-test. **c-e**, Representative images and quantifications for phosphorylated RPA (pSerine 33, **c**), BRCA1 (pSerine 1524, **d**) and RAD51 (**e**) upon estrogen treatment. Similar to 53BP1 foci formation, levels of these protein at DSBs were elevated upon estrogen treatment

In the light of the specific impact of estrogen on ER-positive OvCa cells, we predict ovary-derived epithelial cells are more dependent on HR genes (ie. *BRCA1/BRCA2*) than ER-negative cell types. We first examined the activation of BRCA1 by its phosphorylated form (pS1524), and noticed a ubiquitous phosphorylation signals in OvCa biopsies of both borderline and higher malignant stages (moderately differentiated) (Fig. 3a). A complete negativity in normal ovarian tissues was observed in parallel. Reminiscent of the 53BP1 foci formation in ex vivo culture (Fig. 1b), the ratio of phosphorylated BRCA1 positivity is as high as 85% in cancerous epithelial cells, while the corresponding proliferation rate was only 11% as indicated by Ki67 positivity (Fig. 3b). These observations imply that BRCA1 can be activated in both resting and proliferative OvCa cells, consistent to its dual function in G1 phase (cooperating with MRE11 nuclease) and S/G2 (facilitating resection and committing HR).

Secondly, recruitment of HR factors to DNA lesions in response to estrogen was examined. Higher level of RPA phosphorylation was detected in estrogen-treated OVCAR-8TR cells, indicating a robust generation of single-strand DNA that is required for recombination (Fig. 3c). In consistence, foci formation of RAD51 recombinase and phosphorylated BRCA1 was also increased upon estrogen exposure (Fig. 3d-e), consistent to the S phase response under the same condition (Fig. 2g). Therefore, we conclude that estrogen exposure stimulates homologous recombination in OvCa cells, and that BRCA1 can function beyond HR in both resting and replicating cells.

### Differential requirement of *BRCA* genes by OvCa cells upon estrogen exposure

**Fig. 4:**
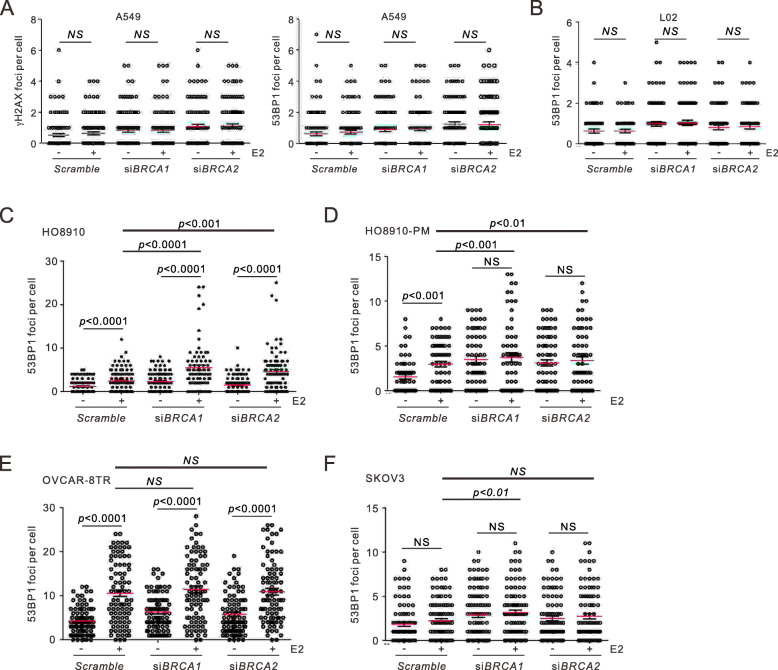
Differential requirement of *BRCA1/BRCA2* by OvCa cells upon estrogen exposure. **a-b**, Quantifications for 53BP1 or γH2AX foci in A549 (**a**) and L02 (**b**) cells after estrogen treatment (50 nM) for 6 h. No elevation of DSBs was observed in these cells. **c-f**, Quantifications for 53BP1 foci formation in HO-8910 (**a**), HO-8910 PM (**b**), OVCAR-8TR (**c**) and SKOV3 (**d**) cells treated for estrogen for 6 h. *p* values: unpaired *t*-test. NS: not statistically significant

To establish the role of HR genes in estrogen-induced genotoxicity in ovarian cancer cells, we examined DSB formation upon combined treatment of estrogen and *BRCA1/BRCA2* ablation in OvCa cell lines from different cancer origins (HO-8910, HO-8910 PM, OVCAR8-8TR and SKOV3), as well as L02 and A549 from non-reproductive tissue. Although depletion of *BRCA1*/si*BRCA2* caused moderate but insignificant increase of 53BP1 and γH2AX in L02 and A549 cells, no additive impact was observed upon estrogen addition (Fig. 4a-b), indicating the irresponsiveness of these non-reproductive cell types due to their low ER expression. In sharp contrast, 53BP1 focal numbers in all OvCa cells but SKOV3 were significantly induced upon estrogen exposure (Fig. 4c-f). When *BRCA1*/*BRCA2* were depleted by RNA interference, all cell lines exhibited 53BP1 elevation relative to scramble transfection.

Intriguingly, *BRCA1*/*BRCA2* silencing caused additive impact on 53BP1 accumulation in sibling HO-8910 and HO-8910 PM cell lines on top of estrogen addition, as shown by the comparison of *Scramble*/E2 versus si*BRCA*/E2 (Fig. 4c-d). This indicates that HO-8910/HO-8910 PM require the function of *BRCA* genes for efficient elimination of estrogen-induced DNA breaks. Unlike its sibling line, HO-8910 PM cells did not display excessive increase of 53BP1 foci upon double treatment relative to si*BRCA1*/si*BRCA2* alone. This could be attributable to its higher ER expression level than HO-8910 (Fig. 2c), conferring a more rigorous response to the basal level of estrogen in culture medium in the absence of *BRCA1*/*BRCA2*.

For OVCAR-8TR cells, estrogen induced more 53BP1 foci upon ablating *BRCA1/BRCA2*, but did not exacerbate the overall number of breaks (Fig. 4e), suggesting that *BRCA* genes do not contribute to repair estrogen-induced DNA damage in OVCAR-8TR. Alternatively, the breaks have reached plateau upon double treatment, considering the median number in OVCAR-8TR is ~ 10 foci per cell compared to 4–5 in other three cells. Taken together, these data consolidate our speculation that HR are required by OvCa cells to remove DNA lesions upon estrogen exposure, though differential responses were observed for individual cell lines.

### Massive genotoxicity in estrogen-treated epithelium of ovary and fallopian tubes

**Fig. 5:**
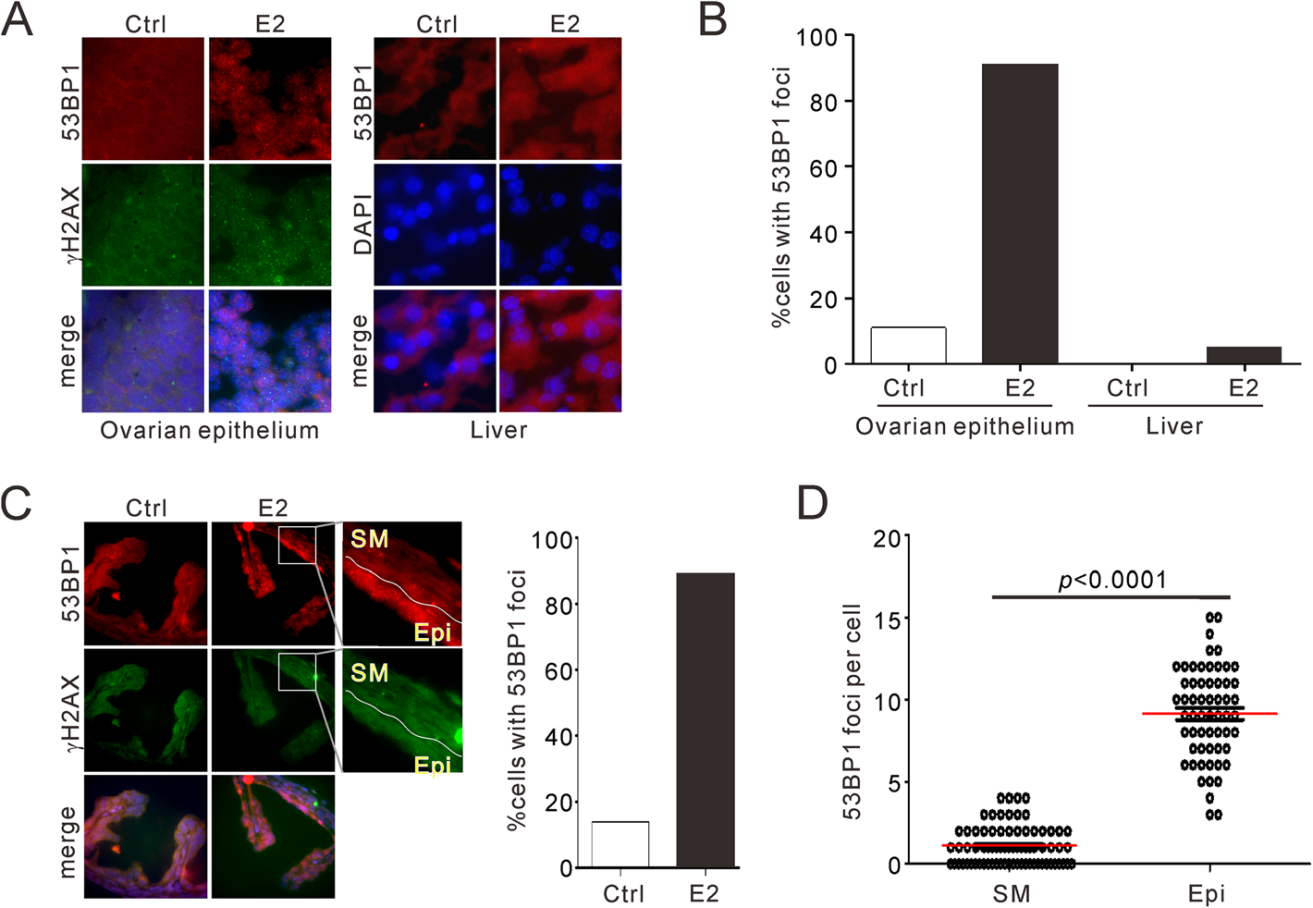
Induction of DNA damage in non-cancerous ovarian and fallopian epithelium upon estrogen exposure. **a-b**, Representative images for 53BP1 and γH2AX foci (**a**) and quantifications for 53BP1 positivity (**b**) in OCT-embedded ovary and liver cryosections. Mice were pre-treated with intraperitoneal injection of estrogen (1 mg/Kg) and sacrificed after 6 h. Dramatic induction of 53BP1/γH2AX foci was observed in epithelial ovary instead of hepatocytes. Pairs of samples from 4 mice were analyzed. **c**, Images (left) and quantification for 53BP1-positive cells (right) for immunostainings for 53BP1 and γH2AX foci in fallopian tube from same mice of (**a-b**). Enlarged areas showing differential foci morphology in epithelial layer (Epi) and smooth muscle (SM). **d**, Comparison for 53BP1 foci number per cell in Epi versus SM compartments. *p* values: unpaired *t*-test between SM and Epi. *n* = 4

It is postulated that OvCa arise from epithelial cells in epithelial compartments of ovary and fallopian tubes [27, 28]. Based on the results described above, we hypothesis that storming of steroid hormones may impair the genomic integrity of non-cancerous cells locating at these pro-carcinogenic sites. To evaluate the in vivo response of ovarian cells to estrogen exposure, we examined the damage responses of epithelial compartments after intraperitoneal injection of estrogen (1 mg/Kg) to mature female mice for 6 h. Indirect immunofluorescent staining of dissected ovarian cryosections showed that a vast number of 53BP1 and γH2AX foci emerged in estrogen-exposed epithelial cells (Fig. 5a). About 86% of ovarian epithelial cells were positive for 53BP1 foci, that is in sharp contrast to the mock-treated ovary and control tissues such as liver (Fig. 5b).

The dramatic DDR response is also observed in fallopian tubes, where similar levels of foci induction were monitored for 53BP1 and γH2AX (Fig. 5c). Intriguingly, we noticed a pronounced cell type specificity in response to estrogen treatment, in that the estrogen-induced foci are nearly exclusively detected in fallopian epithelial monolayer instead of smooth muscle (Fig. 5c-d). This is consistent to the previous detection of *BRCA1* mutation and precancerous lesions in fallopian epithelium [27, 29]. Taken together, we conclude that exposure to estrogen specifically challenges the genome stability of ovarian as well as fallopian epithelium, making HR mechanisms obligatory for eliminating the genotoxicity of these cells.

## Discussion

Genomic surveillance system is a critical anti-cancer barrier considering its function in preventing genomic mutations. Risk for ovarian cancer is remarkably increased in cases losing the DDR genes like *BRCA*1, *BRCA2* and *ARID1A/1B.* It is a long-term mystery how germline *BRCA* mutations predominantly affect female reproductive tissues. In this study, we present evidence that elevation of DNA lesions, corresponding to the ubiquitous activation of BRCA1, is not tightly associated with active genomic replication in OvCa. Our data strongly support the genotoxicity of estrogen can insult genomes of OvCa cells in both G1 and S phase cells, which is highly dependent on the expression of nuclear receptors (ie. ER). Moreover, DNA damage induced upon steroid hormone exposure obligate the function of HR genes as ablation of *BRCA1* and *BRCA2* significantly abrogate the counteraction against estrogen-induced genotoxicity in a subset of OvCa cells.

Although this study mainly involves in vitro work, our data can large reflect the physiological situation of ovarian and fallopian epithelium. Above all, it is difficult to determine the ‘physiological concentration’ of estrogen, due to the natural variation at different menstrual stages, as well as for different tissues (ie. some labs estimated the estrogen concentration in ovary is 100 times higher than other tissues [30]). The in vitro concentration of estrogen corresponding to physiological dosage is estimated for 1 nM [31], and DNA damage could be induced upon exposure to estrogen at this dosage [32]. In this study, we applied higher concentrations of estrogen (50 nM) to visualize DNA damage in OVCAR-8TR cells for the purpose of accelerating the toxic effect of E2 and obtaining quantifiable DNA lesions.

In the light of our in vitro and in vivo data, we conclude that genomic integrity of epithelial compartments in ovarian and fallopian tube is more liable to be challenged by estrogen, relative to non-female reproductive tissues. Considering the expressing level of nuclear receptor and high concentrations of steroids in milieu of ovarian epithelium, the genotoxicity of estrogen would generate strong mutagenic effects in ovarian epithelium. Particularly, given the requirement of HR for eliminating estrogen-induced DNA damage, pathogenic mutations in *BRCA1*, *BRCA2* and *ARID1A/1B* would dramatically exacerbate the mutagenic potential of periodical hormone challenge.

The observation of prevalent induction of DNA breaks and BRCA1 activation/phosphorylation regardless of proliferation status and cell cycle phases indicates a form of replication-independent genotoxicity. This phenomenon is reproduced in OvCa cell lines, primary cancer culture, as well as non-cancerous murine ovarian and fallopian epithelium. Combining the potential of genotoxicity upon estrogen exposure, we conclude that both normal ovarian epithelium and OvCa cells can rigorously respond to high-concentration of estrogen in a ER dependent manner. The genomic insult by steroid hormone is significant, considering the long-term and monthly attack and relatively high dosage of damage (5–15 DSBs per cell in cell culture and normal epithelium), which is equivalent to 0.5–1 Gy of ionizing radiation.

Although we failed to monitor BRCA1 phosphorylation in estrogen-treated murine ovarian epithelium, possibly due to antibody specificity, our data implicate that BRCA1 participates in preventing damage accumulation in both replicating and non-replicating cells in cancer tissue (Fig. 3a). It is likely that BRCA1 cooperates with MRE11 to dispose Top2 adducts in G1 phase, but functions with BRCA2 and RAD51 in S/G2 phase to facilitate HR in removing breaks caused by replication-transcription collisions. Thus, the role of *BRCA1* upon estrogen challenges exceed the HR mechanism, which is also supported by different mutational processes revealed by distinct patterns of genomic imprints born by *BRCA1* and *BRCA2*-mutated cancers [22]. Nevertheless, we conclude that multiple functions of *BRCA1* in counteracting estrogen-induced genotoxicity reflect its central role in obviating the genomic instability of ovarian epithelium and thus disease progression of OvCa.

## Conclusion

Altogether, our study discloses a mechanistic clue for the tissue-specific impact of HR deficiency on ovarian cancers. To counteract the genotoxicity of hormone storming to ovarian and fallopian epithelial cells, HR is activated and loss of this function predetermine the cancer susceptibility in female organs.

## Supplementary information

**Additional file 1 Supplemental Figure 1.** Original data for Fig. 2c showing PCR amplification for ER (left) and actin (right). **Supplemental Figure 2.** Test of siRNA efficiencies in indicated cells. Semi-quantitative PCR (A) and Western blotting were applied to verify the gene silencing of *BRCA1* and *BRCA2* genes. GAPDH amplification was used as internal control. (DOCX 498 kb)

## Data Availability

All data generated or analyzed during this study are included in this published article, and original data can be requested from C.L.

## Abbreviations

OvCa: Ovarian cancer
DDR: DNA Damage Response
DSB: Double strand break
ATM: Ataxia-Telangiectasia mutated
ATR: A-T-related
HR: Homologous recombination
NHEJ: Non-homologous end joining
HRD: Homologous recombination deficiency
DAPI: 4,6-diamidino-2-pheny-lindole
E2: Estradiol
TAM: Tamoxifen
HU: Hydroxyurea
ER: Estrogen receptor
