# Supplementary material for: Cell type-specific genotoxicity in estrogen-exposed ovarian and fallopian epithelium
Source: BMC Cancer. 2020 Oct 21;20:1020. doi: 10.1186/s12885-020-07524-7 (PMC7579787; doi:10.1186/s12885-020-07524-7)

**Supporting information**

**Supplemental Figure 1**

Original data for Figure 2C showing PCR amplification for ER (left) and actin (right).


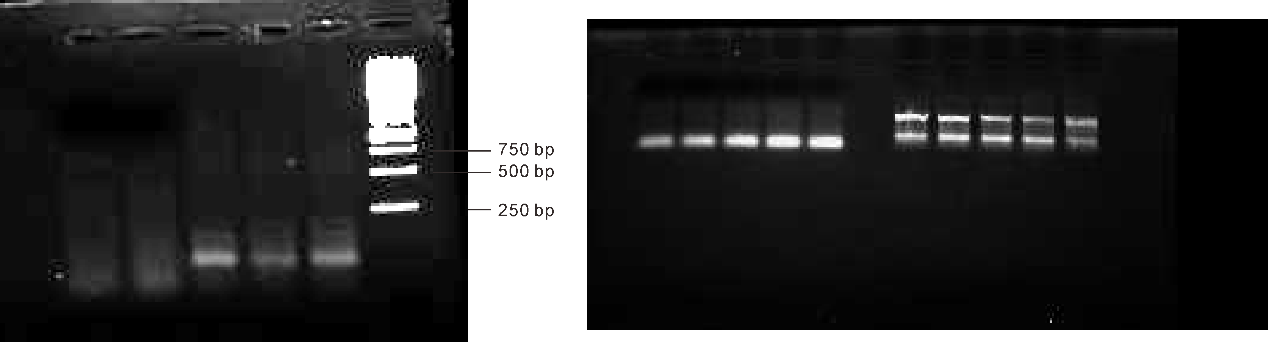


**Supplemental Figure 2**

Test of siRNA efficiencies in indicated cells. Semi-quantitative PCR (A) and Western blotting were applied to verify the gene silencing of *BRCA1* and *BRCA2* genes. GAPDH amplification was used as internal control.


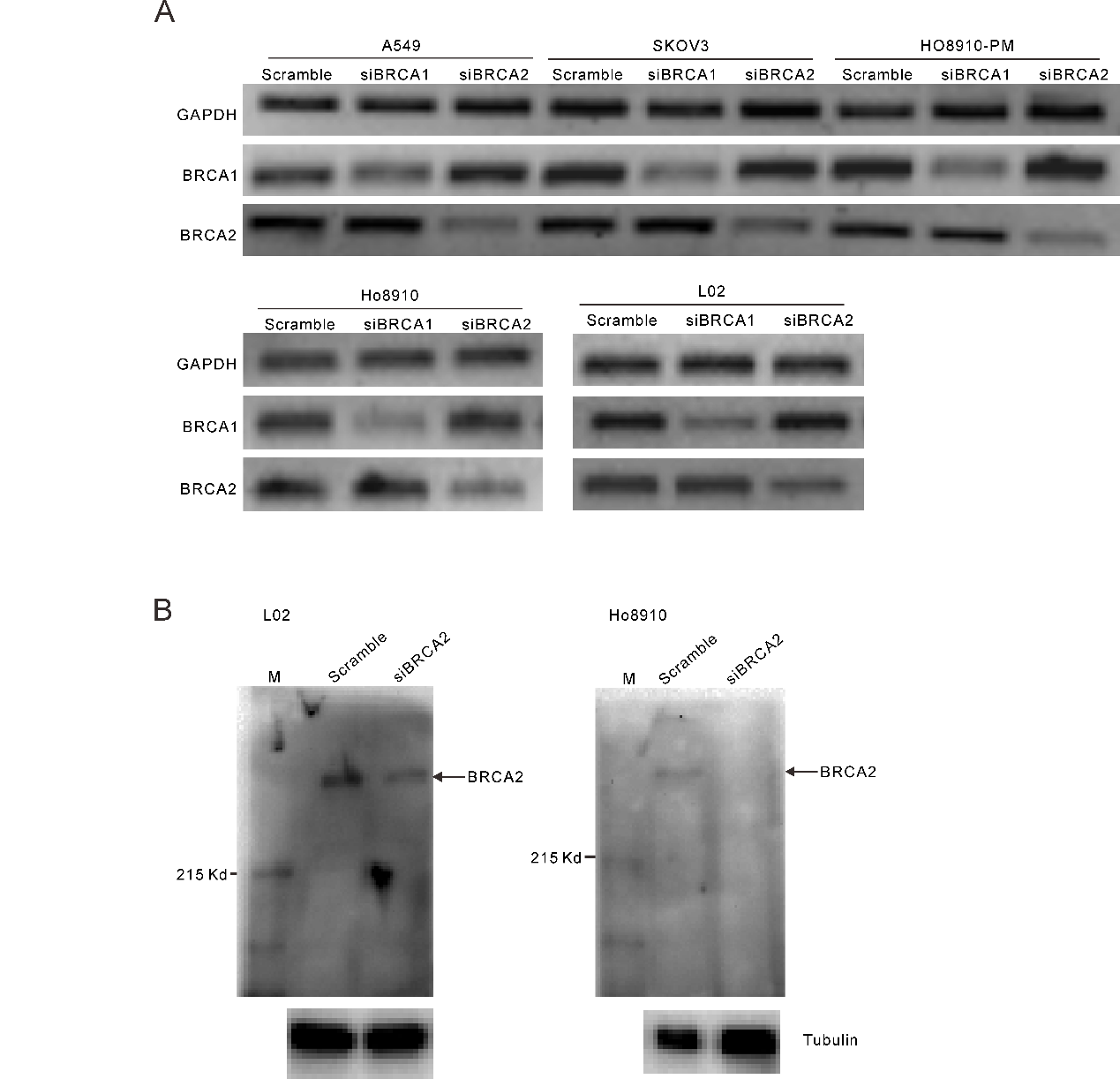

Supplement: Supplementary file 1 — Additional file 1 Supplemental Figure 1. Original data for Fig. 2c showing PCR amplification for ER (left) and actin (right). Supplemental Figure 2. Test of siRNA efficiencies in indicated cells. Semi-quantitative PCR (A) and Western blotting were applied to verify the gene silencing of BRCA1 and BRCA2 genes. GAPDH amplification was used as internal control. (DOCX 498 kb) [file 12885_2020_7524_MOESM1_ESM.docx]
